# Cerebellum, or Not Cerebellum, That Was the Question

**DOI:** 10.7759/cureus.111345

**Published:** 2026-06-23

**Authors:** Sammya Bhattacharya, Dipra Dattasarma, Souvik Sen, Ritwika Paul, Ananya Mukherjee

**Affiliations:** 1 Internal Medicine, KPC Medical College and Hospital, Kolkata, IND

**Keywords:** atypical stroke presentation, brainstem stroke, cerebellar ataxia, pontine infarction, pontocerebellar pathways

## Abstract

Pontine infarction is a recognized subtype of posterior circulation stroke and can present with diverse clinical manifestations. It most frequently involves the anteromedial and anterolateral regions supplied by branches of the basilar artery. Clinical manifestations vary according to the anatomical boundaries of the affected perfusion territory and may present with an atypical constellation of signs and symptoms. We report an atypical case of acute left-sided upper pontine-pontomesencephalic infarction, presenting with severe sensory loss and cerebellar ataxia in a postmenopausal Indian female patient with a background of type 2 diabetes mellitus and hypertension. This case highlights that cerebellar features may manifest in the absence of a primary cerebellar lesion, particularly when such features are bilateral, emphasizing the importance of careful neuroanatomical localization.

## Introduction

The pons, an ectodermal derivative of ventral metencephalon, is located infratentorially in the posterior cranial fossa, situated between the medulla oblongata inferiorly and the midbrain superiorly. It constitutes the largest component of the brainstem and serves as a critical conduit for multiple ascending and descending neural pathways [1,2,3].

The vascular supply of the pons arises predominantly from branches of the basilar artery within the posterior circulation, including the paramedian perforating arteries and short circumferential pontine arteries, with additional contributions from the anterior inferior cerebellar artery (AICA) and superior cerebellar artery (SCA) [1,3,4].

Pontine infarction results from disruption of this vascular supply and represents the most common subtype of posterior circulation stroke, accounting for approximately 40%-50% of such cases. It is most frequently localized to the anteromedial and anterolateral regions supplied by basilar artery branches [5,6].

The underlying etiologies of pontine infarction most commonly include small vessel (branch artery) disease (~30-35%), large artery atherosclerosis (~20%-25%), and cardioembolic events, with hypertension and diabetes mellitus being the most significant predisposing risk factors [7-12].

Clinically, pontine infarction demonstrates a wide spectrum of presentations depending on the arterial territory involved and the anatomical structures affected. Classical syndromes typically involve ipsilateral cranial nerve deficits with contralateral motor and/or sensory impairment. However, atypical presentations, including pure motor hemiparesis, pure sensory stroke, or mixed sensorimotor syndromes, are not uncommon.

Based on arterial anatomy, pontine perfusion territories are broadly classified into four regions: anteromedial, anterolateral, lateral, and posterior [5]. The clinical manifestations correlate closely with the involvement of specific neural structures within these territories.

Clinical presentation is essentially determined by the arterial territory and anatomical boundaries of the region involved by infarction.

At the level of the superior pons, the medial structures from posterior to anterior include the medial longitudinal fasciculus, central tegmental tract, medial lemniscus, corticospinal tracts, corticobulbar tracts, and pontocerebellar fibers, along with pontine nuclei. At the level of the mid-pons, the medial longitudinal fasciculus, medial lemniscus, corticospinal, corticobulbar, and corticopontine tracts are present medially from posterior to anterior. At the inferior pontine level, key structures include the abducens (VI) nerve nucleus complex, medial longitudinal fasciculus, facial (VII) nerve nucleus, medial lemniscus, and corticospinal and corticobulbar tracts [2,3,8].

Given this dense anatomical organization, even small infarcts within the pons can produce complex and sometimes misleading clinical syndromes, making precise neuroanatomical localization essential for accurate diagnosis.

## Case presentation

A 60-year-old Indian woman with a known history of type 2 diabetes mellitus and systemic hypertension presented at the emergency with complaints of deviation of the face to the right, slurring of speech associated with weakness of the right side of the body, and a tendency to lose balance and fall towards the right side on standing, evolving over a duration of two days. Further questioning revealed a history of developing difficulty in swallowing liquids more than solids, a slow and strained low-pitched voice, and difficulty performing fine-coordinated actions. The patient was able to close both eyes and raise both eyebrows. No documented history of loss of consciousness, seizure, headache, vomiting, fever, or other infections, changes in vision, shortness of breath, palpitation, chest pain, cold exposure, or recent upper respiratory tract infection was present. She had never experienced any similar episode earlier. There was no reported history of any recent or old trauma, travel, or usage of illicit drugs. Her family history was non-contributory.

At presentation, the patient was conscious and cooperative, afebrile, and maintaining a blood pressure of 140/80 mm Hg, a heart rate of 84 beats per minute (bpm), and oxygen saturation of 95% in room air. Findings upon examination are presented in Table 1.

**Table 1:** Summary of clinical findings

Domain	Findings
Consciousness	Alert and conscious
Higher Mental Functions	Preserved
Cranium & Spine	No obvious deformities noted.
Intracranial Pressure	No clinical signs of raised intracranial tension.
Cranial Nerves	Left-sided upper motor neuron (UMN) facial nerve (cranial nerve VII) palsy with deviation of the angle of the mouth to the right; mild weakness of the left buccinator and platysma; decreased prominence of the left nasolabial fold; bilateral forehead creases present; able to close both eyelids. Corneal reflex afferents intact bilaterally; facial sensations intact; jaw jerk brisk; gag reflex preserved; no squint or diplopia; no tongue deviation, atrophy, or fasciculations; no tinnitus.
Motor System	Tone: mildly increased in both the upper limb flexors and lower limb extensors. Power: 5/5 in left limbs and 4/5 in right limbs (proximal and distal). Coordination: impaired bilaterally. Pronator drift: negative. No involuntary movements or muscle atrophy.
Sensory System	Exteroception intact. Proprioception: impaired on the right side (posterior column involvement); joint position sense reduced. Cortical sensations preserved.
Reflexes	Deep tendon reflexes diminished in all four limbs; plantar response: right extensor, left equivocal; abdominal reflex absent.
Cerebellar Functions	Nystagmus absent; intention tremor present bilaterally (++); finger–nose test impaired bilaterally, worse with eyes closed; pendular knee jerk present bilaterally (+); dysdiadochokinesia present (+); reeling gait (++); scanning speech moderate; titubation absent.
Stance & Gait	Unable to stand unsupported; wide-based gait; Rombergism present; Romberg’s sign positive.
Autonomic Functions	Not impaired.
Peripheral Nervous System	Within normal limits.
Trophic Changes	No trophic changes noted.

The possibility of intracranial haemorrhage was ruled out with a non-contrast CT scan of the brain. Laboratory investigations did not show any significant dyselectrolytemia. Conservative management was initiated with oral antihypertensives, antiplatelet, statin combination, insulin injection, physiotherapy, swallow therapy, and speech therapy.

Relevant radiological imaging and other investigations revealed the following findings, as described in Figures 1-3 and summarized in Table 2.

**Figure 1:**
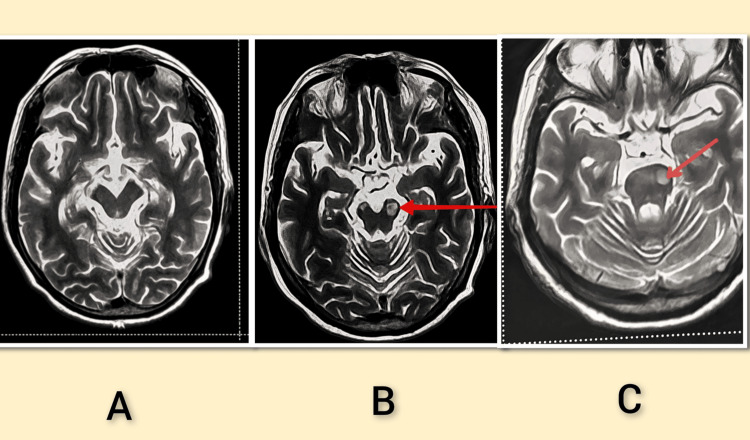
Contiguous axial T2-weighted MRI sections demonstrating a focal hyperintense lesion within the left upper pontine–pontomesencephalic region (indicated by the red arrow), consistent with acute infarction. The lesion appears well-defined without significant mass effect or hemorrhagic transformation. Surrounding brainstem structures are preserved. No corresponding cerebellar parenchymal abnormality is visualized, supporting a brainstem localization despite prominent cerebellar signs clinically. Findings suggest disruption of brainstem-cerebellar pathways, which may have contributed to the cerebellar manifestations observed clinically. (A) Inferior contiguous section below the lesion level demonstrating no focal abnormality. (B) Index lesion section demonstrating focal hyperintensity within the left upper pontine–pontomesencephalic region ( Red arrow). (C) Superior contiguous section demonstrating persistence of the lesion within the upper pontine–pontomesencephalic region (red arrow) and illustrating its relationship to adjacent mesencephalic structures. No focal cerebellar parenchymal lesion is identified in the visualized sections.

**Figure 2:**
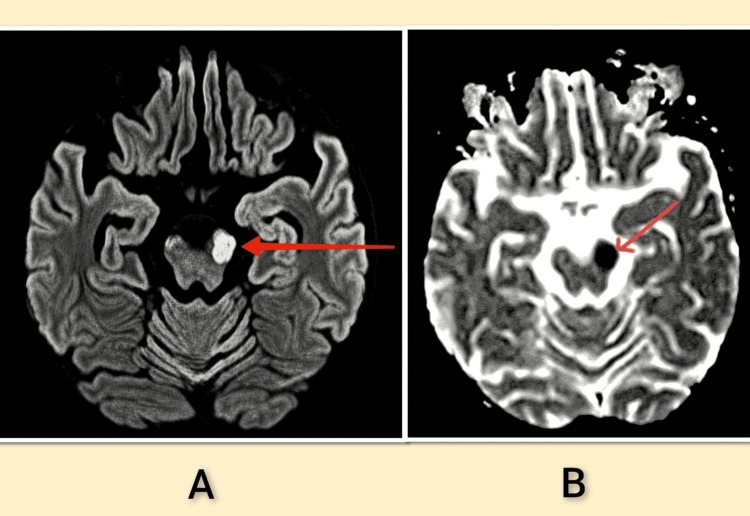
Axial diffusion-weighted imaging (DWI) (A) and the corresponding apparent diffusion coefficient (ADC) map (B) demonstrate an acute infarction involving the left upper pons at the pontomesencephalic junction (red arrows). Corresponding ADC hypointensity supports true restricted diffusion and is consistent with acute ischemic infarction.

**Figure 3:**
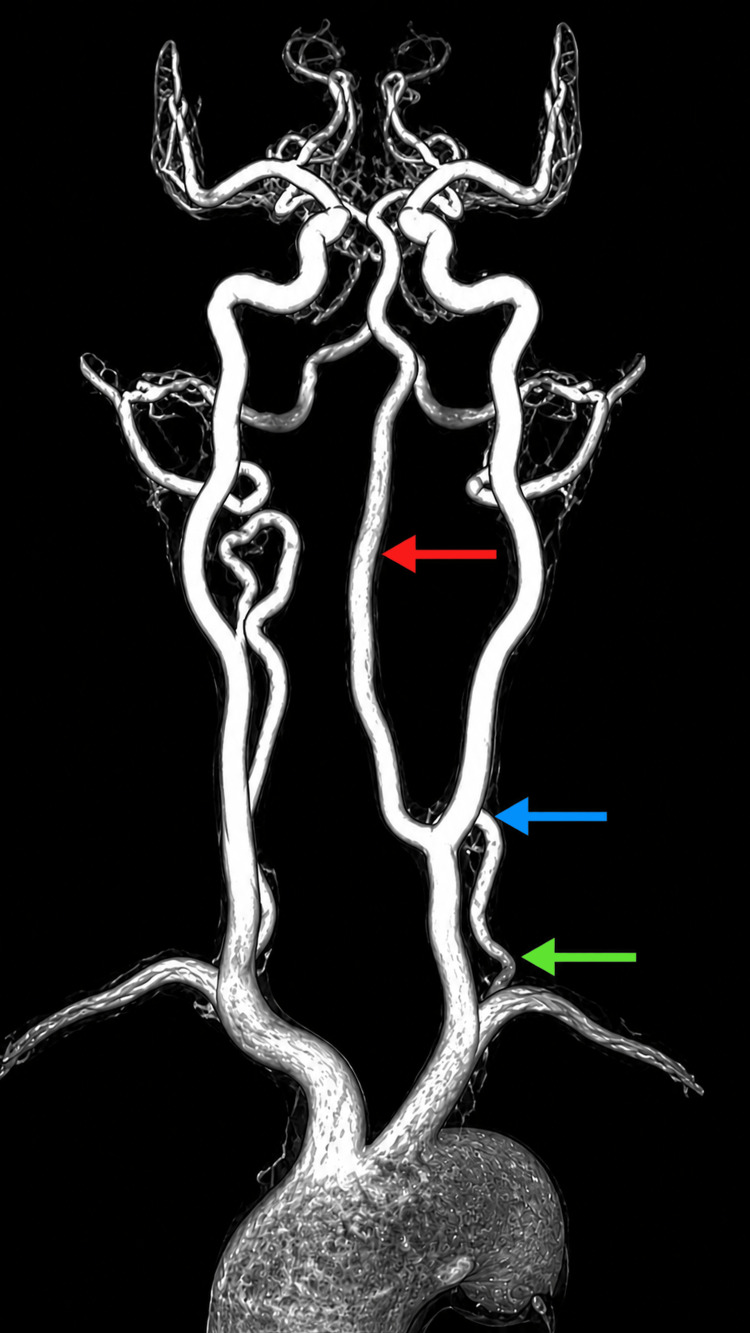
MR angiography showing vertebrobasilar circulation with key cerebellar arterial branches. Three-dimensional MR angiographic reconstruction demonstrates the vertebral arteries converging to form the basilar artery (red arrow) in the midline. From the lower vertebrobasilar system, the posterior inferior cerebellar artery (PICA) arises from the vertebral artery (green arrow), coursing posterolaterally to supply the inferior cerebellum. The anterior inferior cerebellar artery (AICA) (blue arrow) originates from the proximal basilar artery, extending laterally toward the anterior-inferior cerebellar surface and lateral pons. The visualized arterial configuration is consistent with normal vertebrobasilar anatomy without obvious focal stenosis or aneurysmal dilatation.

**Table 2:** Summary of radiological and other investigations findings

Investigation	Findings
12-Lead Electrocardiogram (ECG)	Sinus rhythm; within normal limits
Transthoracic Echocardiography (TTE)	Left ventricular ejection fraction (LVEF): 69%; Grade I left ventricular diastolic dysfunction (LVDD); no regional wall motion abnormality (RWMA); normal left ventricular dimensions; preserved biventricular systolic function; no pulmonary arterial hypertension (PAH); inferior vena cava (IVC) normal
Magnetic Resonance Imaging (MRI) of the Brain (Plain)	Acute non-hemorrhagic infarct involving the left upper pontine–pontomesencephalic region; leukoaraiosis/microangiopathic changes in bilateral periventricular white matter and centrum semiovale; mild bilateral cortical atrophy
4. Magnetic Resonance Angiography (MRA) of the Neck Vessels	Normal study

During hospitalization, the patient remained hemodynamically stable without neurological deterioration. Dysarthria and dysphagia improved gradually with speech and swallow therapy. Gait instability and cerebellar manifestations showed partial improvement with physiotherapy. No new focal neurological deficits developed during this period. After a hospital stay of 10 days, the patient was discharged with single antiplatelet and statin therapy. Glycemic control was addressed with short-acting insulin injection thrice daily, 14 units each before breakfast and lunch, and 12 units before dinner, combined with long-acting basal insulin injection, 20 units once daily before bedtime. The patient was advised to continue physiotherapy, swallow and speech therapy, and strictly adhere to a salt-restricted diabetic diet.

## Discussion

The patient’s clinical presentation allows for precise neuroanatomical localization through careful correlation of signs. Right hemiparesis with right-sided upper motor neuron (UMN) facial palsy typically suggests a left-sided supratentorial lesion. However, the coexistence of left-sided UMN and right-sided weakness with right-sided motor involvement (as seen in our patient) localizes the lesion specifically in the upper dorsal or tegmental pontine-pontomesencephalic region above the level of the facial nucleus. In such cases, involvement of corticospinal and corticobulbar tracts (after they have crossed but before synapsing with the ipsilateral facial nerve nucleus) results in contralateral hemiparesis along with ipsilateral UMN-type facial palsy [1,2]. This condition is also known as pontine supranuclear facial palsy.

The presence of right-sided posterior column sensory impairment further refines localization. The dorsal column-medial lemniscus pathway decussates at the level of the nucleus gracilis and nucleus cuneatus in the medulla and ascends contralaterally as the medial lemniscus. Therefore, ipsilateral loss of proprioception indicates involvement of the contralateral medial lemniscus, supporting a lesion in the left upper brainstem region [2, 3, 4].

Thus, the combined involvement of corticospinal tracts and medial lemniscus strongly suggests a lesion within the left upper brainstem involving the upper pontine-pontomesencephalic region. This constellation of findings is consistent with infarction in the paramedian territory supplied by perforating branches of the basilar artery [5, 6].

A particularly striking feature in this case is the presence of bilateral cerebellar signs, including impaired coordination, dysdiadochokinesia, Rombergism, lateropulsion, and scanning speech. True bilateral cerebellar involvement would typically require lesions affecting both cerebellar hemispheres. However, in upper brainstem infarction, such findings can be explained by disruption of the pontocerebellar pathways. These fibers originate from pontine nuclei and project to the contralateral cerebellar hemisphere via the middle cerebellar peduncle. A strategically located upper brainstem lesion can, therefore, interrupt bilateral cerebellar connections, producing pseudo-cerebellar signs in the absence of a primary cerebellar lesion [1, 7].

The involvement of corticospinal tracts accounts for the increased tone and extensor plantar response observed on the right side, while corticobulbar tract involvement explains the dysphagia, particularly for liquids [1, 8].

Certain aspects of the presentation warrant further consideration. The initial increased tone on both sides and diminished deep tendon reflexes in all four limbs are not entirely explained by a unilateral upper brainstem lesion. These findings may be attributable to chronic microvascular changes, as evidenced by bilateral periventricular and centrum semiovale white matter hyperintensities (leukoaraiosis) on imaging. Such changes, commonly associated with long-standing hypertension and diabetes mellitus, are known to contribute to small vessel ischemic disease and may modify clinical presentation [9, 10].

Additionally, the absence of sixth and seventh cranial nerve nuclear involvement suggests that the infarct was relatively small and localized to the upper pontine-pontomesencephalic region, sparing the lower pontine nuclei. This reflects the segmental anatomical organization of the brainstem [2, 8].

From an etiological perspective, although the clinical profile raises the possibility of an embolic event, extensive evaluation, including MR angiography and Holter monitoring, did not reveal a definitive source. In the presence of vascular risk factors such as hypertension and diabetes mellitus, small vessel (branch artery) disease remains the most likely mechanism [6, 7, 12]. 

Several limitations also merit consideration. MRI also demonstrated chronic microangiopathic white matter changes and mild cerebral atrophy. These pre-existing abnormalities may have reduced neurological reserve and potentially amplified the clinical manifestations of the acute infarction. Therefore, the observed bilateral cerebellar signs may represent an acute-on-chronic presentation rather than being attributable solely to the focal upper brainstem lesion.

## Conclusions

This case reinforces that patients presenting with cerebellar signs and symptoms may not necessarily harbor a primary cerebellar lesion, particularly when such features are bilateral. Instead, underlying brainstem pathology, especially involvement of the upper pontine-pontomesencephalic region, should be carefully considered. This underscores the importance of meticulous neuroanatomical localization and a high index of suspicion to identify subtle yet clinically significant deviations from classical presentations.
